# An Update on Pharmaceutical Strategies for Oral Delivery of Therapeutic Peptides and Proteins in Adults and Pediatrics

**DOI:** 10.3390/children7120307

**Published:** 2020-12-19

**Authors:** Nirnoy Dan, Kamalika Samanta, Hassan Almoazen

**Affiliations:** Department of Pharmaceutical Sciences, University of Tennessee Health Science Center, Memphis, TN 38163, USA; ndan@uthsc.edu (N.D.); ksamanta@uthsc.edu (K.S.)

**Keywords:** oral delivery, proteins, peptides, bioavailability, permeation enhancers, mucoadhesive polymers, enteric coating, cell-penetrating peptides, FDA approved orally administered proteins and peptides

## Abstract

While each route of therapeutic drug delivery has its own advantages and limitations, oral delivery is often favored because it offers convenient painless administration, sustained delivery, prolonged shelf life, and often lower manufacturing cost. Its limitations include mucus and epithelial cell barriers in the gastrointestinal (GI) tract that can block access of larger molecules including Therapeutic protein or peptide-based drugs (TPPs), resulting in reduced bioavailability. This review describes these barriers and discusses different strategies used to modify TPPs to enhance their oral bioavailability and/or to increase their absorption. Some seek to stabilize the TTPs to prevent their degradation by proteolytic enzymes in the GI tract by administering them together with protease inhibitors, while others modify TPPs with mucoadhesive polymers like polyethylene glycol (PEG) to allow them to interact with the mucus layer, thereby delaying their clearance. The further barrier provided by the epithelial cell membrane can be overcome by the addition of a cell-penetrating peptide (CPP) and the use of a carrier molecule such as a liposome, microsphere, or nanosphere to transport the TPP-CPP chimera. Enteric coatings have also been used to help TPPs reach the small intestine. Key efficacious TPP formulations that have been approved for clinical use will be discussed.

## 1. Introduction

**Figure 1:**
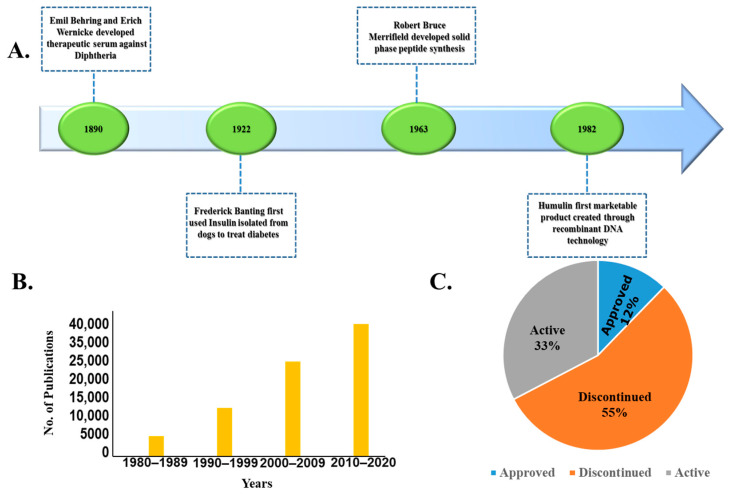
The burgeoning field of protein- and peptide-based therapeutics. (**A**): Key developments in the field; (**B**): number of publications on “oral delivery of peptides and proteins” in PubMed, arranged by decade from 1980 to the present; (**C**) Status of clinical trials of peptides and proteins.

Currently, small molecule drugs (<900 Daltons) dominate the pharmaceutical drug market. However, recent advances in recombinant DNA technology and solid-phase synthesis of peptides and proteins have enabled large-scale production of therapeutic peptides and proteins (TPPs) [1]. The success of recombinant human insulin as a protein-based therapeutic for the treatment of diabetes mellitus, the first commercially available recombinant protein approved by the U.S. Food and Drug Administration, revolutionized the field of TPPs (Figure 1A) [2]. Over the next three decades, the development of TPPs grew significantly in both academia and industry (Figure 1B,C), with the number of FDA-approved TPPs reaching 239 by 2017 [3]. As of March 2020, the FDA’s Center for Drug Evaluation and Research (CDER) had approved 96 Biologics License Applications (BLAs). According to the report by Zion market research, the global peptide-based therapeutics market was estimated at approximately USD 23.1 billion in 2017 and anticipated to reach around USD 43.3 billion by 2024. The global oral proteins and peptides market is estimated at around USD 643 million and likely to grow around $8233 million by 2028 [3,4].

From a clinical perspective, TPPs are inherently more specific to their target sites than small molecule drugs, resulting in reduced interference with the patient’s normal physiology and consequently in fewer off-target effects.

Another advantage of using proteins that are produced naturally in the body for therapeutic applications is that they tend to be less immunogenic, producing fewer side effects due to inadvertent stimulation of the immune system. From a financial standpoint, median total pre-market development times are shorter for biologics (10.6 years) than for small molecule drugs (12.6 years), as estimated by the Merck Index. In 2009, the US Congress passed the Biologics Price Competition and Innovation Act (BPCIA) which gave newly approved biologics 12 years of guaranteed exclusivity [5]. Thus, the road to the commercial market is comparatively easier for biologics compared to small molecule drugs [6].

The most commonly utilized routes of administration for TPPs are intravenous (I.V), intraperitoneal (I.P.), and intramuscular (I.M.) injections. However, oral administration is the route most preferred by patients as it avoids the pain and discomfort associated with injections. Furthermore, oral medications are less expensive to produce than medications that must be isotonic and sterile to use for injection and cheaper to administer since they require no specialized equipment or trained professionals [7]. However, the development of orally available dosage forms of TPPs have been complicated by their high molecular weight, possible hydrophilicity, poor stability in physiological conditions, short biological half-life, and low permeability through the epithelial barrier in the small intestine [8]. An ideal oral delivery platform for TPP drugs should retain their therapeutic activity and protect them from the proteolytic enzymes and acidic environment found in the stomach, before releasing their active forms in the neutral environment of the intestine to get absorbed into the bloodstream. In this review, we will discuss the challenges associated with the oral delivery of TPPs and ongoing efforts to overcome them.

## 2. Gridlocks of Oral Delivery of Peptides and Proteins

### 2.1. Enzymatic Barriers of the Digestive System

**Figure 2:**
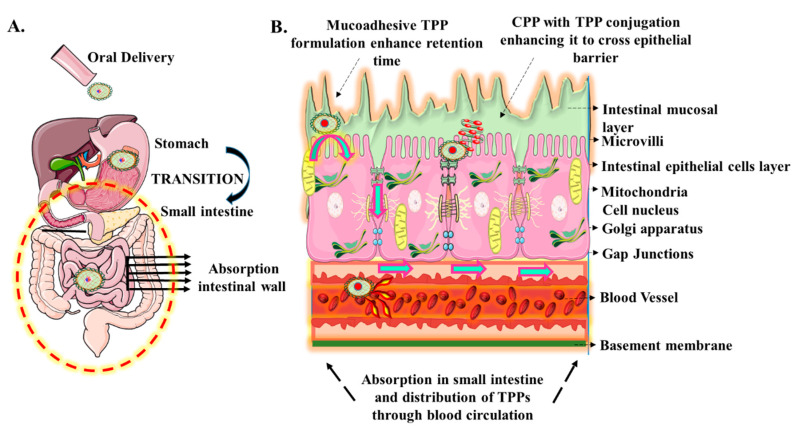
Oral delivery of drugs and their absorption into the bloodstream. (**A**): Path of orally administered therapeutic peptide and proteins (TPP) through the digestive system; (**B**): key components and pathways for absorption through the intestinal epithelial barrier.

Orally administered TPPs follow the same path as ingested food in their passage through the G.I. tract (esophagus, stomach, small intestine, and large intestine), before being absorbed into the bloodstream from the small intestine [9]. Therefore, an understanding of the anatomy and physiology of the different compartments of the G.I. tract is crucial for the successful design of oral dosage formulations of TPPs. In the mouth, ingested food or drug is mixed with saliva, which helps in the smooth movement into the esophagus. Salivary enzymes such as amylases and lipases aid in the digestion of starch and fats but the degradation of proteins in the mouth and esophagus are minimal [10]. In the stomach, the acidic environment (pH 1.5–3.5) is maintained by gastric acid (consisting of hydrochloric acid, potassium chloride, and sodium chloride) secreted from oxyntic cells (Figure 2A) [11]. While gastric acid destabilizes protein and peptide structure and exposes peptide bonds for enzymatic degradation by the enzyme pepsin, the majority of enzymatic degradation of proteins occurs in the small intestine.

Proteolytic enzymes promote the digestion of peptides and proteins into their constituent amino acids. This is accomplished primarily by pepsin, trypsin, chymotrypsin, and elastase enzymes produced by the pancreas and by the mucosal cells that line the gut [12,13,14]. Peptidases in the microvilli of the intestinal epithelial cell brush border membrane such as aminopeptidase and dipeptidyl peptidases 3 and 4 digest peptides of up to 10 amino acids, while intracellular peptidases are specific for dipeptides [14,15,16,17]. As with all proteins, TPPs are susceptible to degradation by proteolytic enzymes in the stomach and small intestine, which complicates their delivery by the oral route. However, formulations of TPPs that use nanoparticles, microparticles, liposomes, or an enteric coating to encapsulate them serve to protect them from degradation, as discussed further in a later section [5].

Several factors affect the proteolysis of proteins in general, including molecular weight, hydrophilicity, the potential for ionic or hydrogen bond formation, the number of enzyme susceptible groups present, and low structural flexibility. For example, peptides that lack a cyclic structure and small peptides fewer than 12 amino acids in length (desmopressin, oxytocin, Arg-vasopressin, octreotide, and cyclosporin) are less readily digested by proteases than proteins or larger peptides containing more than 12 amino acids (somatostatin, calcitonin, glucagon, secretin, and insulin) and having more complex structures [18]. The amino acids produced by enzymatic degradation and other nutrients released by digestion of food are absorbed into the bloodstream from the small intestine, facilitated by the tremendously large surface area provided by the mucosal folds, villi, and microvilli. Absorption is very negligible in the large intestine. The large intestine hosts ~700 species of bacteria, which aid in digestion and absorption of the residual food that arrived from the small intestine [19]. However, it’s neutral to slightly alkaline pH (pH range 6–8), the absence of proteolytic enzymes, and the extended residence time make the large intestine an exciting target for future delivery of TPPs.

### 2.2. Mucus and Epithelial Barriers of the Intestine

The low bioavailability of orally administered TPPs is linked to their reduced ability to cross the epithelial lining in the small intestine and its protective mucus layer. Appropriate knowledge of the structure of intestinal epithelium and absorption mechanisms at intestinal mucosa can help in engineering delivery vehicles for TPP to overcome this barrier (Figure 2B). The intestinal epithelial barrier consists of a single layer of columnar epithelial cells supported by the lamina propria and muscularis mucosae layers. The epithelial cells are connected by tight junctions, forming a functionally contiguous membrane that is covered by a highly glycosylated mucus layer [16]. The mucus is secreted from the intestinal goblet cells and gastric foveolar cells. It is comprised of water (95%) and mucin, an O-linked glycoprotein with oligosaccharide side chains and terminal sialic acid and sulfate groups that give it a net negative charge [20,21]. This unstirred water layer restricts both passive and active diffusion of solutes [22]. Although mucus mesh space (20–200 nm) is large enough to allow diffusion of some smaller macromolecules such as human papillomavirus (HPV; 55 nm), Norwalk virus (38 nm), and small globular proteins, it effectively traps larger macromolecules and blocks their access to the underlying epithelial cells [23]. A periodic turnover every 4–6 h. renews the mucus layer, leading to the rapid clearing of entrapped nanoparticles or proteins [24].

The use of mucoadhesive polymers for the delivery of drugs like TPPs relies on hydrophobic, hydrogen bonding, and Van der Waals interactions between the polymer and the mucin protein. The first successful strategy to use nanoparticles as drug delivery particles involved coating 200–500 nm (diameter) nanoparticles with a dense layer of a muco-resistant, low molecular weight polyethylene glycol (PEG) polymer [25]. Its mucus-inert properties, the hydrophilicity of the coating polymer, and a net neutral charge helped these nanoparticles to evade entrapment in the mucus layer [26]. Subsequent researchers have developed cell culture-based assays and purified mucin preparations for the evaluation of various compounds as drug delivery vehicles that can successfully cross the mucus layer [27,28].

Once through the mucus layer, the epithelial cell layer still blocks access to the bloodstream. Although mucous penetrating nanoparticles do facilitate the passage of drugs through the mucous barrier, their densely charged hydrophilic surface prevents them from entering the epithelial cells [29,30]. More recently, epithelial cell entry of insulin has been achieved using a cell-penetrating peptide and coupled with a coating of the mucus-inert polymer pHPMA. The success of this strategy relies on the disassociation of pHPMA after crossing the mucus barrier and the cell-penetrating peptide facilitating transport across the epithelial cell membrane [31].

However, entry into epithelial cells still may not ensure the absorption of the drug into the bloodstream. The apical side of the epithelial membrane contains efflux pumps that can return the drug back into the lumen of the gut, thereby contributing to multidrug resistance in humans [32]. One such efflux pump is P-glycoprotein-I, also known as multidrug resistance protein I (MDRI), which returns linear and cyclic peptides, including cyclosporine, to the lumen after epithelial cell entry [15].

### 2.3. Transport across the Epithelial Cell Membrane

The single-cell epithelial barrier can be crossed via three different mechanisms, namely paracellular transport, passive transcellular transport, and active carrier-mediated transcellular transport (Figure 2B). Paracellular transport involves the movement of solutes through spaces between epithelial cells by passive diffusion, a process regulated by structures called tight junctions (*zonulae occludens*) [33]. However, since tight junctions typically allow the passage of molecules no larger than 22–30Å in diameter, large molecules like proteins (>100–200 Da) are generally excluded from paracellular transport [34,35,36,37]. The size and charge of TPPs would generally exclude them from paracellular transport.

Transcellular transport can be mediated by either passive diffusion or actively by specific amino acid, peptide transporter, or endocytosis while paracellular transport takes place via passive diffusion only [38]. The transcellular pathways transport-specific solutes such as ions, amino acids, sugars, and short-chain fatty acids into or through epithelial cells, often using transporters specific for each type of cargo. Most oral drugs are absorbed passively by the transcellular pathway, in part due to the large surface area of the brush border membrane available for absorption, which is 1000 times larger than the paracellular surface area [39]. Permeability studies in cultured human intestinal epithelial Caco-2 cell monolayers showed preferential transport of hydrophobic and higher molecular weight compounds by the transcellular rather than the paracellular route [40]. During transcellular transport, TPPs are imported across the brush border apical membrane following a diffusion gradient, move through the cell to the basolateral membrane, and, with the help of specific active carrier transport proteins, move into the bloodstream by facilitated diffusion [41]. Transcellular transport of TPPs is dependent on their size, shape, charge, hydrophobicity, molecular structure, and partition coefficient [42].

## 3. Protein and Peptide Delivery for Pediatric Use

Most TPPs including vaccines are administered to children using a needle and syringe. The oral route is the most convenient way of administering medicine in children as if offers a painless approach. However, oral delivery of medicines including TPPs in children remains challenging as many of the conventional strategies do not work on this age group. Children also show different responses to many active ingredients and excipients than do adult patients. Pediatric formulations need special consideration depending on the developmental age of the child. For instance, a Danish study shows that children less than 6 years of age have difficulties in swallowing solid dosage forms like tablets. Parents report that unpleasant taste and swallowing are a huge problem for solid dosage forms. Therefore, a liquid formulation with acceptable taste is preferred for this age group as they are more readily swallowed [43]. The dose can be easily modified in liquid formulations based on the body weight and surface area. Most of the drugs (~90%) for children are liquid formulations administered via the oral route.

Children’s anatomy and physiology change very rapidly with increased age, especially in the developmental ages (1–1.5 years) and these changes have a direct impact on the pharmacokinetic profile of any formulations [44]. The International Conference of Harmonization (ICH) has divided pediatric patients into five different groups based on the physiological differences between different subsets. Pharmacokinetics parameters of a formulation in children differ from adults due to differences in physiological parameters like gastric pH, transit time, gastrointestinal motility and permeability, reduced surface for drug absorption, low renal clearance, and reduced activity of drug-metabolizing enzymes (Table 1). For example, gastric emptying rate, intestinal transit time, and surface area for absorption are reduced in newborns and neonates compared to those in adults (Table 1). A recent focus on understanding the anatomy and physiology of children and develop successful formulation strategies tailored for specific age groups. Most of the formulation for pediatric oral delivery utilizes different strategies like the incorporation of permeation enhancers, enzyme inhibitors, and surfactants, as described below. However, the choice of any pharmaceutical excipient in the formulation must consider potential pediatric toxicity. Commonly used excipients like benzyl alcohol, dyes, propylene glycol, and dextran are safe for use in adults but are toxic in pediatric patients [45]. While propylene glycol passes the blood-brain barrier and causes brain damage, benzyl alcohol can cause a gasping syndrome in children, which can be fatal in neonates and infants [46].

In this section, we will discuss some novel formulation approaches for pediatric oral drug delivery. Conventional tablets are slow to disintegrate and children may find them difficult to swallow. Orodispersible tablets rapidly disintegrate in the mouth when they come in contact with saliva or water, thereby easing the process of swallowing, and can be taken without water [51]. These formulations can be further modified with taste-masking agents, flavoring agents, and sweeteners to enhance patient compliance. This platform is suitable for delivering drugs with good aqueous solubility. The most obvious component for this formulation is a disintegrant. Cellulose derivatives like carboxymethyl cellulose and sodium starch glycolate are commonly used as a disintegrant in orodispersible tablets [52]. Conventional methods of processing like freeze-drying, lyophilization, and direct compression or molding result in tablets that lack fast disintegrating properties. Alternative strategies for orodispersible tablet formation include Orasolv^®^, a marketed tablet formulation manufactured by a conventional compressing method but that incorporates taste-masking and effervescent agents [53]. Orodispersible films with the same advantages as orodispersible tablets are also emerging as a tool for pediatric drug delivery. They use a polymer matrix to disintegrate very quickly in the mouth, releasing the active ingredient. It possesses an elegant appearance that may be preferred by some patients. Orodispersible films can be manufactured by conventional solvent casting, hot-melt extrusion methods, or even 3D inkjet printing technologies [54]. Orodispersible films can be tailored for buccal administration of protein and peptides in children or, if delivery via the oral cavity is not appropriate, can be formulated to protect the drug from the harsh conditions of the GI tract. Orodispersible films can also be used to deliver potent drugs as a very small amount of drug loading (60–70 mg) can be achieved. To date, no clinical success has been achieved with such films in children. Development of proper orodispersible strategies for TPP delivery may be a time-consuming process as it will entail developments in the manufacturing process and toxicity-compatibility studies to identify safe excipients for use in children.

## 4. Strategies for Enhancing Oral Delivery of Therapeutic Peptides and Proteins

### 4.1. Permeation/Absorption Enhancers

One way to improve the bioavailability of TPPs in the circulation is to increase their ability to cross the mucus and/or epithelial cell layers to reach the bloodstream [55]. However, the disruption of these barriers must be selective and transient to prevent unwanted entry of pathogens or toxins [16]. This can be accomplished using permeation enhancers (Pes), either co-administered with the TPP or incorporated into their structures. These elements can variously interact with the epithelial cell membrane lipid or proteins, open or destroy the integrity of the tight junctions, lower mucus viscosity, or increase membrane fluidity. Many candidate compounds have been investigated as potential absorption enhancers but were proven to be toxic, leading to significant, irreversible damage to the gastrointestinal mucosa [56]. Successful permeation enhancer activity depends on the time to onset of action, the degree of permeation enhancement, and the absence of toxicity [57]. Several recognized classes of compounds (chelating agents, surfactants or detergents, bile salts, and fatty acids and their salts) serve as successful permeation enhancers, each with its own mechanism of action (Table 2).

Chelating agents such as ethylenediaminetetraacetic acid (EDTA) and ethyleneglycoltetraacetic acid (EGTA) modulate or perturb structural integrity of tight junctions by chelating calcium ions essential for their structural organization. This disruption increases the paracellular transport of TPPs. Calcium chelating agents can be toxic when calcium depletion induces distortion of actin filaments, adherent junctions, and cell adhesion molecules [33].

Surfactants (detergents) destabilize proteins and lipid present at membranes and increase intercellular space for transportation of molecules like TPPs. These include anionic detergents such as Tween 20, sodium dodecyl (lauryl) sulfate (SDS; also known as sodium lauryl sulfate), and sodium dioctyl sulfosuccinate and non-ionic detergents such as polysorbate 80 and polyoxyl 40. Anionic surfactants are more effective than non-ionic surfactants in increasing transepithelial permeability [58]. Other soluble surfactants include the alkyl maltosides dodecylmaltoside (DDM) and tetradecyl maltoside (TDM). TDM increases oral bioavailability of the anticoagulant drug enoxaparin by transiently reducing transepithelial electrical resistance in C2BBel cell extracts. Within 60 min of TDM treatment, the normal transepithelial electrical resistance is restored [59].

Bile salts such as sodium glycocholate and sodium taurocholate represent another class of surfactants anchor at the lipid bilayer of the epithelial cell membrane and form micelles above or near the critical micellar concentration (CMC), thereby eliminating the membrane lipid components [60]. The bile salt sodium deoxycholate increases paracellular absorption of hydrophilic drugs and both transcellular and paracellular absorption of hydrophobic drugs by reducing the transepithelial electrical resistance in Caco-2 cell monolayers [61]. Bile salts also increase the oral bioavailability of salmon calcitonin and insulin [62]. Sodium taurodeoxycholate successfully improved oral absorption of epidermal growth factor peptide both in cultured human Caco-2 cells and in a xenograft model [62,63,64].

Medium-chain fatty acids (FAs) function as absorption enhancers by a mechanism that is not well understood. They are hypothesized to downregulate tricellulin and claudin-5 expression, thereby affecting the integrity of the tight junctions, and to solubilize the epithelial cell plasma membrane phospholipid bilayer reversibly [65]. Orally administered dispersions of insulin with saturated fatty acids such as lauric, palmitic, and stearic acids as PE reduced blood glucose levels when administered to rabbits [66]. Sodium caprate, a sodium salt of the medium-chain FA capric acid that is an FDA-approved food additive has been tested as an absorption enhancer for oral delivery of insulin in conjunction with an analog of glucagon-like peptide 1 (GLP-1), using Gastrointestinal Permeation Enhancing Technology (GIPET™; Merrion Pharmaceuticals, Dublin, Ireland) [65,67]. At a 10 mM concentration, sodium caprate induces rapid and reversible opening of tight junctions, allowing paracellular transport and bloodstream uptake of proteins and peptides up to 10 kDa [68].

Other molecules can also function as PEs. Zonula occludens toxins (ZOT), produced by the bacterium *Vibrio cholerae* reversibly increase paracellular absorption of orally administered insulin in rabbit intestine 10-fold via enhancing the permeability through the tight junctions without toxicity issues [69]. ZOT only acts through a specific intestinal receptor reversibly and inactively in microflora containing colon, which is susceptible to mucosal damage. A biologically active carboxyl-terminal fragment of ZOT (12 kDa) enhances intestinal absorption of the drugs susceptible to efflux transporters by opening the tight junctions and interacting with PGP [70].

### 4.2. Structural Modification

The structure and function of proteins are tightly linked, particularly for enzymes that must maintain the conformation of an active site to retain enzymatic activity. Alteration of TPP structure can improve stability and membrane penetration, decrease immunogenicity, and improve resistance to proteolytic degradation. Such alterations can occur by modification of accessible amino acid side chains, by alteration of the carbohydrate moieties of glycoproteins, or by conjugating them to lipophilic molecules such as fatty acids to increase their hydrophobicity [71]. For example, conjugation of insulin to palmitic acid derivatives (monopalmitoyl-insulin and dipalmitoyl-insulin) resulted in lower blood glucose levels and less immunogenicity than that observed for native insulin [72]. Similarly, the addition of lauric acid to the N-terminal glutamyl group of thyrotropin-releasing hormone (TRH) resulted in a form of THR that was stable in the mucus layer, taken up efficiently by the epithelial cells in the brush border, where it was converted into its native form [73].

Another approach is the introduction of protective groups that prevent enzymatic degradation of TPPs. The addition of α-lipoic acid to insulin protects it from digestion by trypsin [74]. Similarly, the incorporation of ethoxycarbonyl methionine into insulin protected the amino groups of glycine and lysine residues, resulting in an insulin derivative that remained intact even after 5 cycles of Edman degradation in vitro [75]. Modification of the antidiuretic hormone vasopressin by deamination of the N-terminal amino acid and substitution of D-arginine for L-arginine near the C-terminus produced desmopressin. These changes enhanced passive transport of the desmopressin over that of vasopressin, possibly due to protection from proteolytic degradation [76]. Finally, cyclization of TPPs can increase their half-lives by protecting them from the digestion of exo- and endo-peptidases in the bloodstream. Since flexible structures serve as better substrates for proteolytic enzymes and cyclization of peptides and proteins generate more rigid conformations, cyclized TPPs exhibit decreased susceptibility to enzymatic degradation [77].

### 4.3. Enzyme Inhibitors

An alternative to structural alteration is the co-administration of protease inhibitors like pancreatic inhibitor, soybean trypsin inhibitor, camostat mesylate, and aprotinin, which increase the bioavailability of orally administered TPPs by reducing their enzymatic digestion by trypsin or α-trypsin [78]. Protease inhibitors can be classified as (i) amino acid-based and modified inhibitors (e.g., N-acetylcysteine), (ii) Peptide or modified peptide inhibitors (e.g., bacitracin, cyclic dodecapeptide), (iii) Polypeptide protease inhibitors (e.g., aprotinin), or (iv) non-amino acid-based inhibitors (e.g., disisopropyl fluorophosphate and phenylmethylsulfonyl fluoride (PMSF)) [79]. The appropriate choice of enzyme inhibitor depends on the location and cellular distribution of the target enzyme. For example, inhibitors of luminally secreted proteases such as aprotinin differ from those that inhibit membrane-bound proteases, such as amastatin and bestatin. Non-amino acid-based inhibitors like disisopropyl fluorophosphate and phenylmethylsulfonyl fluoride (PMSF) inhibit not only serine proteases but also the potent neurotransmitter acetylcholinesterase, which makes them toxic and unacceptable for pharmaceutical use [79]. By contrast, the non-amino acid inhibitor FK-448 exhibits low toxicity while inhibiting chymotrypsin and serves to increase intestinal absorption of insulin in rats and dogs [80].

Other protease inhibitors such as chymostatin, Bowman–Birk inhibitor, and aprotinin increase the bioavailability of orally administered insulin microspheres by preventing degradation by digestive enzymes like pepsin, trypsin, and α-chymotrypsin [81]. The high molecular weight of polypeptide protease inhibitors like aprotinin allows their efficient formulation in the sustained release oral dosage forms such as insulin-loaded polyvinylalcohol-gel spheres [82]. Aprotinin is one of the pioneering enzyme inhibitors used in several oral TPP formulations because it inhibited trypsin and chymotrypsin [83].

Two assays have been used to evaluate the protective effects of these inhibitors. One test for measuring luminally-secreted protease activity is to incubate the TPP delivery system with the protease inhibitor in the gastric or intestinal fluid at 37 °C and then determining the amount of residual substrate drug in the medium [84,85]. For in vivo protease inhibition studies, insulin is used as a model drug because it is degraded by both luminal and brush border proteases [81,85]. Enzymatic inhibitors have significantly contributed to enhancing the bioavailability of orally administered TPPs. However, the use of protease inhibitors can lead to inactivation or overactivation of unintended cellular enzyme activities, leading to severe side effects like unstable digestion of food and hypertrophy or hyperplasia of the pancreas [86]. Therefore, care is required in choosing a protease inhibitor that does not cause off-target effects.

### 4.4. PEGlyation

Another alternative strategy for the protection of TPPs from proteases is to covalently attach them to a biocompatible polymer, leading to reduced immunogenicity, enhanced bioavailability, and enriched pharmacokinetic profile. Polymers such as PHPMA (poly(N-2-hyfroxypropyl methacrylamide)), POEGMA (polyoligo(ethylene glycol) methyl ether methacrylate), PNIPAm (poly(N-isopropylacrylamide)), PLGA (poly(lactic-co-glycolic acid)), PDEAM (poly(N,N-diethylacrylamide)); and PEG (polyethylene glycol) are used to facilitate oral delivery of TPPs. PEG exhibits several advantages over other polymers of the same molecular weight, including biocompatibility, lower toxicity, improved biological and circulation half-life, and lower cost [87]. PEG is amphipathic, i.e., it is soluble in both polar and non-polar solvents [88]. PEG can be derivatized with a functional group to allow chemical conjugation with reactive amino acids of proteins or peptides, resulting in PEGylated derivatives [89]. PEGylation protects the structural integrity of TPPs by shielding them from proteolytic enzymes, allowing them to reach the epithelial lining and the bloodstream beyond.

PEGylation also serves to decrease the immunogenicity and immunotoxicity of TPPs in vivo [90]. Conjugation to PEG increases the size of small peptides, reducing their ready excretion by glomerular filtration. Several parental PEG-modified TPP formulations are FDA approved for oral delivery in humans and others have been investigated in human clinical trials [91,92]. A recombinant human granulocyte colony-stimulating factor (hG-CSF)-PEG complex increases its stability and retention in vivo [93]. An insulin-PEG delivery system with a thiolate polymer poly(acrylic acid)–cysteine carrier matrix was more stable in vitro and more effective in reducing blood glucose levels in diabetic mice when administered orally than was native insulin [94]. An oral insulin formulation, hexyl-insulin monoconjugate 2 (HIM2), conjugated to PEG and a PE via an amide bond at Lys-β29 has been tested for its ability to treat type I diabetes mellitus. A single oral administration of HIM2 (0.75 mg/kg) increased the whole-body glucose disposal and inhibited endogenous glucose production in healthy nondiabetic human subjects in a dose-dependent manner [95]. HIM2 is effective in treating both type 1 and type 2 diabetes [96,97]. HIM2 was jointly developed by NOBEX Corporation and BIOCON; it is approved for use in India under the brand name Insugen [78].

### 4.5. Mucoadhesive Polymeric Systems

**Figure 3:**
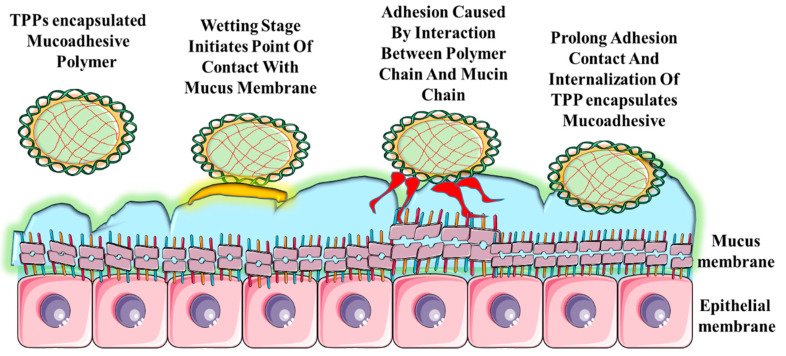
Steps in mucoadhesion for the mucoadhesive polymeric system. At the first stage the mucoadhesive polymer swells (contact stage). The swelling occurs because the polymer has an affinity for water. In the second stage, the polymer interacts with the mucus membrane. Weak chemical bonds form in the entangled polymer chains.

Mucoadhesive polymers such as hydroxypropyl methylcellulose, chitosan, and Carbopol^®^ (Lubrizol Corporation, Wickliffe, OH, USA) that adhere to the mucus layer in the G.I. tract provide an excellent platform for oral delivery of TPPs. Mucoadhesion enhances the degree of drug absorption by extending the residence time of the drug carrier and providing higher drug concentration to the absorption site (Figure 3). Hydrophilicity and high molecular weight of the mucoadhesive polymers enhance the solubility of poorly absorbed peptides and proteins while protecting them from enzymatic degradation in the G.I. tract [83]. A chitosan-coated nanosphere comprised of elcatonin significantly reduced blood calcium levels when compared to the uncoated nanosphere-elcatonin solution in rats [98]. N-trimethyl chitosan chloride showed enhanced absorption compared to normal chitosan salts in neutral and basic conditions [99].

The properties of these polymers dramatically change in response to changes in the environment, including pH, temperature, ionic strength, and the presence of certain enzymes. For example, PAA (polyacrylic acid) or PMA (polymethacrylic acid) hydrogels with azo-aromatic crosslinkers have been developed for targeted delivery of macromolecules to the colon. The swelling of these polymers and release of their cargo occurs only with an increase in pH and is highest in the neutral pH of the colon. However, the enzyme azoreductase, produced by microbial flora in the colon, can degrade azo aromatic linkers [100]. Another example of a stimuli-sensitive mucoadhesive polymeric system is presented by insulin-loaded microparticles (100–150 μM) constituted of PMA- and polyethylene glycol (PEG)-crosslinked P(MAA-g-EG) (poly(methacrylic-g-ethlyene glycol) hydrogel polymer network. The P(MAA-g-EG) polymer network inhibits calcium-dependent proteolytic enzymes such as trypsin [101]. It protects insulin from the acidic environment of the stomach as the polymer network only swells and releases its cargo in the neutral and basic environment of the intestine. The PEG molecules provide adhesive properties that allow them to adhere to the epithelial cell layer [102]. Orally administered insulin loaded microparticles showed good pharmacokinetic properties and hypoglycemic effect both in healthy and diabetic rats and these effects last up to 8 h. after administration [102].

Incorporating a biocompatible endogenous permeation enhancer such as spermine or spermidine improved the bioavailability of calcitonin-loaded mucoadhesive PAA nanoparticles, which. showed a significant and prolonged reduction in blood calcium levels when administered orally in rats. This polyelectrolyte nanoparticle complex was made from the interaction of the carboxylate groups of the PAA and magnesium ions or the amino acid groups of the spermine [103].

The gastrointestinal mucoadhesive patch system (GI-MAPS) was developed for oral administration of TPPs, including granulocyte colony-stimulating factor (G-CSF). GI-MAPS is comprised of four distinct layers, each serving different functions, and contained in an enteric coating. The base layer contains a water-insoluble ethylcellulose polymer that protects the protein cargo from proteolytic enzymes. The surface layer is comprised of an enteric pH-sensitive polymer like hydroxypropylmethylcellulose phthalate (HP-55^®^), Eudragit L100^®^, or Eudragit S100^®^, covered by an adhesive compound. The middle layer is comprised of a cellulose membrane containing the drug of interest, either alone or coupled with substances like citric acid and a non-ionic detergent, polyoxyethylated castor oil derivative (HCP-60^®^). This unique system was initially formulated for oral delivery of recombinant human G-CSF in dogs and resulted in a slight (1.3-fold) increase in total white blood cells [104].

Thiolated mucoadhesive polymers contain thiol groups able to form disulfide bonds with the cysteine groups of mucus glycoproteins, thereby increasing their mucoadhesive properties, while also serving to protect the TPP cargo from enzymatic digestion [105]. When thiolated chitosan polymers and unmodified chitosan were each labeled with the passive diffusion marker rhodamine 123, the thiolated chitosan exhibited a 3-fold higher permeation rate than the unmodified chitosan [106]. High MW thiomers are more mucoadhesive due to significant entanglement of mucus glycoproteins with the long-chain polymer but they suffer from reduced mobility and poor penetration across the mucus layer [107]. Low MW, short-chain thiomers show higher penetration and mobility but negligible entanglement with the mucus glycoproteins [108]. Combining both high and low MW thiomers in a single preparation resulted in better gelling and mucoadhesive properties [109]. Although mucoadhesive polymers improved absorption of coupled TPPs, their application is of limited duration due to the mucus turnover process that occurs in the human intestine every 12–24 h [106].

### 4.6. Cell-Penetrating Peptides

**Figure 4:**
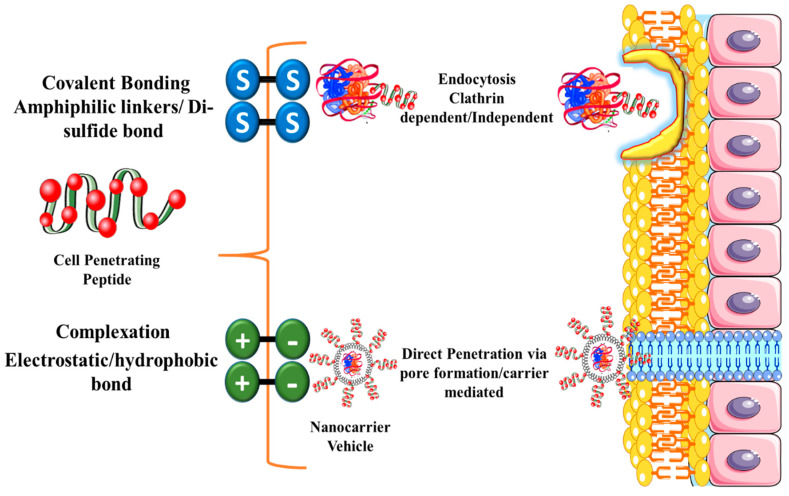
Conjugation and uptake mechanism of Cell-Penetrating Peptide mediated TPPs. Cell-Penetrating Peptides can be conjugated to the TPP either via covalent bonding using amphiphilic linkers, disulfide bonding, or by complexation using electrostatic interactions, hydrophobic bonding. Cell-Penetrating Peptides are up taken generally by two mechanisms (endocytosis or direct penetration via pore formation).

The low bioavailability of TPPs can also result from the failure of hydrophilic peptides and proteins to cross the hydrophobic epithelial membrane [110,111,112]. Another strategy used to improve oral delivery of TPPs is to couple them to 3–30 amino acid cell-penetrating peptides (CPPs), either via covalent bond formation (disulfide or amide bonds or specific linkers) or physical (electrostatic or hydrophobic) interactions [8,113], as shown in Figure 4. CPP-mediated delivery can occur by its direct fusion with the lipid bilayer of the epithelial membrane or by endocytosis by epithelial cells [66].

The exact mechanism of CPP action can depend on the sequence, concentration, stability, and stereochemistry [114]. For example, in cultured MC57 fibrosarcoma and HeLa cells, the L form of amino acid-based CPP shows increased uptake relative to the D form of the same amino acid [115]. Uptake of CPPs can be enhanced by changing amino acid stereochemistry or by modifying amino acid groups at specific sequences to avoid excess cleavage caused by proteases. When used to direct delivery of human calcitonin to HEK 293 T cultured cells, the substitution of N-methyl-phenylalanine for D-phenylalanine at a specified position in the protein decreased its proteolytic degradation without altering its penetration properties [116].

The most common CPPs are derived from the HIV-1 (Tat) protein, model amphipathic peptide (MAP), synthetic transportan peptide, and the *Drosophila melanogaster* homeobox protein antennapedia [117,118]. For example, the HIV-1 Tat-derived CPP successfully enhanced the passage of 120 kDa beta-galactosidase protein through the blood-brain barrier [119]. CPPs have also been used to effectively deliver antiviral antisense peptide nucleic acids (PNAs) complementary to the HIV-1 transactivation control region (TAR) into cells in BALB/c mice, either alone or conjugated with CPPs derived from the antennapedia protein (penetratin) or from HIV-1 transcriptional activator TAT protein. When compared to unconjugated PNA-TAR formulation, those coupled to CPPs showed increased uptake, although all three compounds showed similar distribution and clearance profiles in the mice [120].

### 4.7. Buccal Delivery as an Alternative “Oral” Route

Some delivery strategies seek to overcome issues of low bioavailability by avoiding the gut entirely. One such strategy involves the delivery of drugs into the bloodstream in the oral cavity, which avoids their degradation by the gastric acid and proteolytic enzymes present in the GI tract but introduces new challenges in delivering drugs in therapeutically relevant quantities by this route. A greater understanding of the anatomy and physiology of the oral cavity can help in designing suitable delivery strategies that overcome low bioavailability issues.

The oral cavity consists of several parts, including the lips, alveolar mucosa, hard and soft palate, cheeks, gingiva, and tongue. Over sixty percent of the total surface area of the oral lining consists of the lining mucosa. This is comprised of stratified squamous epithelial cell layers, separated by a basement membrane from the underlying lamina propria and submucosa, both of which contain blood vessels. Absorbed drugs traverse through the small capillaries of the lamina propria to the lingual, facial, and retro-mandibular veins that connect with the jugular vein or systemic circulation [121]. The buccal and sublingual mucosa of the cheek and the floor of the mouth (under the tongue), respectively, have been used for drug delivery due to a higher degree of permeability than seen for the other regions [122]. The buccal and sublingual mucosa possess a non-keratinized surface, non-polar lipids, and an amorphous organization of intercellular lipid that provides less resistance to peptide-based drug diffusion via the paracellular pathway. In contrast, heavily keratinized regions like the lips, palates, and gingiva contain a stratum corneum comprised of lipid lamellae with dense interior matrices that are impregnable to any foreign material including drugs [123]. The sublingual route is predominantly used for delivering small molecules with the desired rapid onset of action. By comparison, the buccal route has a smaller surface area and lower permeability and is more appropriate for delivery drugs like TPPs where a longer duration of action is preferable. The major challenges to the successful delivery of drugs via the buccal route are saliva, which can reduce the therapeutic activity of the drug by removing it from the target site, and the activity of metabolic enzymes. Several drugs, including insulin, proinsulin, enkephalin analogs, thyrotropin-releasing hormone, and calcitonin are degraded when incubated with animal buccal tissue homogenates [124]. TPP formulations designed to facilitate their delivery via the buccal route have incorporated enzymatic inhibitors such as bile salts, aprotinin, or polyacrylic acid (PAA) to hinder enzymatic degradation. Several strategies like bio-adhesive tablets, patches, ointments, and powders have been employed to facilitate the buccal delivery of TPPs [125].

## 5. Formulation Approaches

Several strategies have been applied to develop effective vehicles for improved oral delivery of TPPs. These are reviewed briefly here.

### 5.1. Enteric Coatings

Enteric coatings such as pH-sensitive polymers have been used to protect TPPs from proteolysis in the gut and thereby facilitate their delivery to the absorption site in the small intestine. This strategy has been used for the oral delivery of protease-sensitive insulin to the small intestine [126]. Insulin enclosed within gelatin capsules coated with different ratios of the polyacrylamide polymers Eudragit RS, L, and S and delivered orally to rats resulted in maximum insulin release at pH 7.5 and 8 from the formulations containing Eudragit RS1 and RS2, respectively. Measurement of blood glucose in treated rats showed a significant level of hypoglycemia when the formulation when compared with the control [127]. Non-toxic, pH-sensitive methacrylic acid copolymer serves as a stable delivery agent that can survive the acid pH in the gut [128,129]. For example, it has been used to deliver 5-amino-salicylic acid to the GI tract for the treatment of ulcerative colitis [130]. It has also been used to protect insulin from proteolysis [131]. However, because enteric coatings enhance the bioactivity of encapsulated TPPs but do not play any role in enhancing the process of absorption, protease inhibitors and membrane permeability enhancers can be added to enhance the overall efficacy of enterically coated TPP formulations [132].

### 5.2. Liposomes

Lipid-based carrier systems have been widely studied for oral delivery of proteins, but they are inefficient in encapsulation of hydrophilic peptides and proteins, are instable in the GI tract, and exhibit poor permeation through the mucosa to reach the epithelial layer. However, these challenges can be countered significantly by modifying the liposome surface with a mucoadhesive polymer like chitosan and protease inhibitors like aprotinin or the Bowman–Birk inhibitor. Orally delivered achitosan-aprotinin conjugates showed a 15-fold increased AUC (area under curve) compared to calcitonin when administered as a single agent [133]. Another surface-modified mucoadhesive polymer-based liposome formulation is the carbopol-lectin liposome conjugate. When conjugated covalently to calcitonin, the lectin conjugate shows better pharmacokinetic properties and pharmacological effect in vivo than carbopol-calcitonin liposomes lacking lectin [134]. A coating of silica on the liposomes also helps in preventing proteolytic degradation of the encapsulated drug and increase encapsulation efficiency [135]. Fusogenic liposomes containing the envelope glycoprotein from the Sendai virus aids in internalizing cargo proteins into the cytoplasm by membrane fusion. Fusogenic liposome-encapsulated insulins show enhanced insulin absorption, as demonstrated by a rise in hypoglycemic effect with very little mucosal damage [136].

Liposomes can be chemically modified to improve its delivery characteristics. Liposomes can be surface modified to deliver their cargo to a specific delivery site. For example, liposomes made from di-stearoyl phosphatidylcholine, phosphatidylserine, and cholesterol were selectively directed to the Peyer’s patches of the lower ileum of the small intestine [137]. Modifying the liposome surface with PEG increases their half-time of circulation by protecting them from opsonization and capture by the reticuloendothelial system (RES) [138]. Double liposomes that contained an inner core of a smaller liposome (100 nm) within a larger liposome have been used for oral delivery of insulin to diabetic rats. Comparison of four different types of inner liposomes (neutral, cationic, VET, and cationic-VET) showed an enhanced hypoglycemic effect when the cationic charged core was used, due to the ability of the double liposomes to protect its cargo from enzymatic degradation and of the positively charged inner liposome to enhance intestinal absorption [139]. Since liposomes can show physical instability, evidenced by vesicle aggregation, fusion, and creaming during manufacturing and storage, liposomes were coated with a silica layer that provides strong protection at the liquid interface and also enhances in vivo absorption [135]. The source of the lipids that comprise the liposomes can also influence their protective properties. For example, archaebacterial lipids protect proteins from extreme pH, oxidation, and the action of bile salts and lipases [135]. Oral administration of insulin encased in achaeosomes (engineered liposomes comprised of membrane lipids derived from the archaebacterium *Sulfolobus acidocaldari*) is superior to that by traditional liposomes in lowering blood glucose levels.

### 5.3. Microspheres

Polymeric microspheres that range in size from 1 to 1000 μm can also facilitate controlled oral delivery of peptides and proteins [140,141]. Polymeric microspheres have been used in oral vaccine preparations. For instance, the *Vibrio cholerae* whole-cell oral microsphere vaccine relies on the formation of poly(lactic-co-glycolic acid) copolymer (PLGA) microspheres containing a whole cell lysate from *V. cholerae* [142]. Japanese encephalitis virus vaccine was also prepared by encapsulating it in a biodegradable PLGA based microsphere [143]. Another orally administered PLGA microsphere of lactoglobulin was used to treat newborns prone to milk protein allergies. Tween 20 was added in this formulation, which increased protein encapsulation efficiency and superior controlled release [144]. Some proteins show structural malformation when encapsulated in PLGA microspheres by the water-in-oil-in-water (w/o/w) double emulsification method as they can aggregate and their structures can unfold at the water/organic interface. However, these issues can be resolved using alternative solid-in-oil-in-water (s/o/w) or solid-in-oil-in-oil (s/o/o) emulsification methods of encapsulation. Because proteins are stable and rigid in the organic phase, unfolding or other structural modifications does not occur [145].

Insulin loaded microspheres were fabricated from blends of PEG with poly(L-lactide) (PLA) homopolymer and poly(DL-lactide co-glycolide) (PLG) copolymers by water-in-oil solvent extraction method. These particles exhibited controlled release of insulin over 4 weeks, although only the PLA/PEG particles and their insulin cargo were stable over the 4 weeks [146].

Biologically erodible microsphere formulations made with polyanhydride copolymers of fumaric and sebacic acid (poly(FA:SA)) demonstrated better adhesive properties than microspheres made with other polymers and were able to penetrate through the intestinal epithelium and the follicle-associated epithelium covering the lymphoid tissue of Peyer’s patches [147]. Stable pH-sensitive microspheres can also be synthesized inexpensively from chemically modified soybean hydrolysate with aromatic acyl chlorides. These low-cost microspheres were stable at acid pH (<3.5) and soluble at pH values greater than 5.0. Oral delivery of salmon calcitonin or porcine insulin using these microspheres resulted in higher bioavailability [148]. Alginate-chitosan insulin microspheres were synthesized using a membrane emulsification technique combining calcium ions and chitosan polymer solidification with excellent loading efficiency (56%) and activity maintenance (99.4%). These microspheres showed remarkable stability in simulated gastric fluid and reduced blood glucose level successfully in diabetic rats for more than 60 h [149].

### 5.4. Microparticles/Nanoparticles

Microparticles or nanoparticles (10–200 nm) penetrate through the mucus pores, cross the epithelial membrane, and reach the bloodstream. However, larger nanoparticles (200 nm–500 nm) that are surface-modified with PEG also effectively diffusing through the mucus layers [25]. Nanoparticle absorption through the GI tract is governed by the nature of its components, size, and surface charge. Nanoparticle delivery systems have several advantages, including protection from acid and proteolytic enzymes in the GI tract; slow, controlled, and site-specific delivery of the cargo; the ability to cross the mucus layer due to their small size; its high volume and large surface area for interaction with mucus and the epithelial membrane, and ability to deliver cargo via the oral route for improved bioavailability and pharmacokinetics properties [150,151,152]. Polymers like alginate, chitosan, cyclodextrins, and PLGA are used to manufacture nanoparticles for oral administration of TPPs. Polymers can be further modified by grafting or substituting with functional groups that can be used for targeting specific tissues, opening tight junctions, mucoadhesion, or permeation enhancement. Alginate-chitosan nanoparticles for oral delivery of insulin were synthesized by coating alginate (anionic polysaccharide)-containing insulin cores with chitosan (a cationic deacetylated form of chitin). FITC-labeled alginate-chitosan nanoparticles containing insulin show uptake both on the surface of the intestinal enterocytes and M-cells of the Peyer’s patches. Oral administration of these nanoparticles significantly reduced blood glucose levels in diabetic rats [153]. Nanoparticles coated with blood proteins were engineered for site-specific uptake of the cargo by the mononuclear phagocytic system to reduce off-target effects [154]. The nanoparticle surface can also be modified with ligands like VB12, lectins, biotins, or folic acid to facilitate pronounced uptake by receptor-mediated endocytosis [155]. However, one disadvantage of this kind of delivery system is liver toxicity, since the nanoparticles accumulate in the liver.

## 6. Clinical Investigation of Oral Peptides and Proteins

There is much interest in the pharmaceutical industry in exploring methods of oral delivery for TPPs. For example, in Tarsa Therapeutics’ ORACLE trial, salmon calcitonin was delivered orally to osteoporotic postmenopausal women for 48 weeks, resulting in a 1.5% increase in the bone density together with a decrease in serum cartilage breakdown markers when compared with control. This study demonstrated that oral delivery of proteins can be efficacious [156]. Clinical trials of some promising formulations of TPPs with effective oral delivery agents are summarized below.

### 6.1. Eligen^®^ (Emisphere)

Eligen^®^ (Emisphere) is a delivery agent that uses sodium N-(8-(2-hydroxybenzoyl) amino caprylate) (SNAC) as a carrier molecule for attaching drugs through weak non-covalent association. The integrity of the drug remains unchanged when conjugated with the carrier molecule and can be delivered across the epithelial membrane. SNAC enhances insulin absorption 10-fold transcellularly without significantly damaging the tight junctions. It also helps to maintain TPP stability in the GI tract by protecting the coupled TPP/SNAC protein from proteolytic enzymes [150,151]. A phase I clinical trial (NCT00982254) compared the efficacy of oral insulin/SNAC and subcutaneous injection of regular human insulin for the treatment of type 2 diabetes. Under fasting conditions, patients treated with oral insulin/SNAC show higher maximum insulin concentration in plasma and exhibit a faster onset of insulin action, but a high degree of variability in absorption between patients was observed [157]. In a phase II trial, Eligen insulin (together with metformin) failed to achieve significantly better glycemic control than metformin treatment alone [54].

### 6.2. Cross-Linked Enzyme Crystals (CLEC; Altus Pharmaceuticals)

CLEC is a novel platform for the production of protein microcrystals cross-linked with glutaraldehyde, which serves to stabilize protein structure and reduce proteolytic degradation. This approach has been used successfully for lipases, esterase, and the protein hormone calcitonin [158]. Commercially available TheraCLEC™-protease along with TheraCLEC™-lipase are extensively used as pancreatic enzyme products and adjuvant therapy in autoimmune disorders and infectious diseases. Protease- CLEC formulation is also being investigated for the treatment of pain in chronic pancreatitis [158,159].

### 6.3. BioOral (BioSante Pharmaceuticals)

The size and surface area of nanoparticles makes them appropriate vehicles for site-specific delivery of protein cargo. They are nonimmunogenic, biodegradable, easy to modify, easy to metabolize, and stable during storage [160]. The BioOral system has been developed as calcium phosphate-based nanoparticles for facilitating vaccine delivery. Oral delivery of insulin has been formulated as CAP-PEG-Insulin-Casein (CAPIC), where casein encapsulates the insulin-PEG formulation and acts as an enteric coating to protect insulin from low pH in the stomach. It is available in the market from BioSante Pharmaceuticals [161].

### 6.4. Oral-Lyn^TM^ (Generex Biotechnologies)

Oral-Lyn is used commercially for aerosol delivery of insulin delivery. Excipients used for formulating Oral-Lyn are nonchlorofluorocarbon-based propellent and stabilizing agents to protect the aerosolized protein formulation from denaturation. Insulin is converted into its micelle form with a size greater than 7 µm, which is too large to enter the lungs but can reach the bloodstream via the buccal route of oral delivery. Oral-Lyn aerosols are delivered with the RapidMist device also used for the delivery of asthma medications. Clinical data shows that Oral-Lyn in its Phase III has been used for treating Diabetes Mellitus (NCT00668850). Generex has been marketing the same product in India under the name of Oral Recosulin since its launch on 20 November 2008 in a collaboration with Shreya Life Sciences [162,163].

### 6.5. HIM2 (Nobex and Biocon)

Hydrophilic proteins and peptides are difficult to deliver through the oral route. Nobex and Biocon together are trying to address this limitation on a commercial scale by introducing a lipophilic moiety into the protein structure. The hydrophobic nature of the protein would help in increasing its ability to cross the epithelial membrane. Hexyl-insulin monoconjugate (HIM2) is a modified form of insulin where amphiphilic oligomers conjugated with a primary amine group of the Lys-29 residue found in the beta chain of human insulin [97]. It is resistant to enzymatic degradation and improves insulin absorption after oral administration. Ongoing studies on HIM2 shows its use as a glucose stabilizer in type 1 diabetes mellitus [96].

### 6.6. Oraldel^TM^ (ANL010; Apollo Life Science)

Although enteric coatings are successful for the delivery of insulin, proteolysis, and absorption at intestinal enterocytes limit its commercial use [164]. Vitamin B12 binds to a salivary enzyme, hepatocorrin, which transports it to the small intestine, where it is absorbed into the bloodstream. Consequently, the coupling of insulin to vitamin B12 resulted in internalization and transport of insulin to the serum [155]. The Oraldel^TM^ formulation uses sugar nanoparticles coated with vitamin B12 for oral delivery of functional insulin molecules encapsulated in the matrix and can actively normalize blood glucose levels when delivered orally [155,165].

### 6.7. AI-401 (Eli-Lilly and Autoimmune Inc.)

AI-401 is a recombinant insulin formulation developed using oral tolerance therapy, which suppresses autoreactive T cells in type I diabetes mellitus [166]. Phase II clinical trial on this therapy has shown the normal working of endogenous insulin by delaying the destruction of beta islet cells in the pancreas [166]. Clinical studies have been completed in patients suffering from Type I diabetes [167].

### 6.8. Orasome (Endorex Corporation)

Orasome is a polymer-based liposome that is designed to protect insulin and human growth factor from the acidic stomach pH and release it into the small intestine. Orasome also helps in protecting the protein from low pH and bile salts [168,169].

### 6.9. Gastrointestinal Permeation Enhancement Technology 1 (GIPET-1^®^; Merrion Pharma and Novo Nordisk)

GIPET-1^®^ is another oral delivery technology used for enhancing protein absorption by as much as 200 times when delivered orally. This delivers oral therapeutics such as Insulin and GLP-1. GIPET-1 is currently used in tablet form of insulin with three different coatings and has been studied for its pharmacokinetics and pharmacodynamics properties towards the treatment of diabetes mellitus and is currently the subject of a Phase I clinical trial (NCT01931137) [170,171,172].

### 6.10. Oramed (Oramed Pharmaceuticals)

Oramed is also a carrier system used for delivering GLP-1 and Insulin. Ormade’s oral insulin is currently being investigated for both type1 and type2 diabetes. This formulation is available as ORMD-0801, which protects insulin from proteolytic degradation and enhances its oral delivery when passing the G.I. tract. It is under phase II clinical trial when used for insulin delivery and phase I trial when used for GLP-I delivery (NCT02535715) [173,174].

### 6.11. Peptelligence^TM^ (Unigene Laboratories and Enteris Biopharma)

This is an enteric-coated tablet formulation that increases solubility and absorptions of peptides. This formulation has two main components: a surfactant permeation enhancer that loosens the tight junctions and potentiates paracellular transport and a multifunctional pH-lowering citric acid component that increases absorptive flux acts as a membrane wetting agent and is a calcium chelator [175]. This strategy is under different stages for clinical compounds with different TPPs. TBRIATM, an oral calcitonin formulation for osteoporosis based on the Peptelligence^TM^ platform, successfully completed clinical trials and has obtained the approval of its new drug application (NDA) from the FDA [67].

### 6.12. C-Trelin Orally Disintegrated (OD) Tablet (MediForum)

This is an orally degradable drug composed of C-Trelin, an active analog of Thyrotropin-releasing Hormone, which functions by inhibiting glutamate activation and is one among the most successful drugs for the treatment of SCA [176]. Currently, it is under Phase 4 trial in patients with ataxia caused by spinocerebellar degeneration (NCT04107740) [176,177].

### 6.13. QRH-882260 (University of Michigan)

QRH-882260 is an orally bioavailable heptapeptide imaging agent labeled with the fluorophore cyanine 5.5 (Cy5.5) linked to the 7 aa peptide that binds specifically to the epidermal growth factor receptor, EGFR, and is used to image EGFR-expressing tumor cells. Because 97% of adenocarcinomas of the colon overexpress EGFR, this peptide provides an important imaging agent for multiple areas in the human G.I. tract [178]. Currently, this peptide is in its Phase I trial as per the 2018 update (NCT02574858) [179].

### 6.14. (STAMP)C16G2 Antimicrobial Peptide (Armata Pharmaceuticals, lnc.)

This is a targeting antimicrobial peptide developed as an oral gel treatment of dental caries. It specifically targets the cariogenic oral pathogen *Streptococcus mutans* without interfering with other strains of *Streptococcus* [180]. Its G2 domain effectively targets the pathogen membrane, leading to membrane disruption, subsequent leaking of small molecules, and cell death. It is currently under Phase II clinical trial (NCT02509845) [181].

### 6.15. Oshadi Icp (Oshadi Drug Administration)

Oshadi Icp is a carrier system developed for oral delivery of a combination of insulin, proinsulin, and C-peptide. This formulation was developed and tested as OR14-1 [182]. It enables complete absorption of intact proteins from the GI tract for the treatment of type I diabetes mellitus. A successful outcome of this system would avoid the multiple daily injections required for insulin therapy [182]. A Phase II clinical trial has been completed (NCT01772251) [183].

### 6.16. Oral Recombinant Salmon Calcitonin (rsCT; Tarsa Therapeutics)

RsCT is a 32-amino acid naturally-occurring calcium regulating peptide that can bind to G-protein receptors expressed on osteoclasts, leading to inhibition of nonapoptotic osteoclastic activity. rsCT is a tablet formulation of orally available calcitonin primarily used in women for preventing bone loss and treatment of low bone density for avoiding the risk of fracture. The oral form of calcitonin might have higher efficacy than compared to the nasal form of its administration based on the ORACAL trial research [156]. A Phase II trial has been completed (NCT01292187) [184].

### 6.17. Oral hPTH (EBP05; Entera Bio Ltd.)

Oral hPTH is a synthetic parathyroid hormone therapy for the treatment of hypoparathyroidism that targets eucalcemia and converts quiescent bone to high-turnover bone [185]. A Phase II clinical trial involving oral administration of hPTH showed promising results on serum calcium levels and a reduction in serum phosphate and urinary calcium levels (NCT04003467) [185,186,187].

### 6.18. Oral Semaglutide (Novo Nordisk)

Semaglutide is a glucagon-like peptide 1 receptor agonist (GLP-1 RA) drug for type II diabetes Miletus that plays an important role in lowering hemoglobin A1c, but its sub-cutaneous administration has caused a loss in its therapeutic efficacy. Oral administration may prevent drug disintegration in the stomach, thereby facilitating its transcellular absorption via the gastric membrane, allowing it to reach the systemic circulation. A Phase III clinical trial is completed (NCT02906930) and the FDA approved it for treatment of type 2 diabetes [188,189,190].

### 6.19. Oral OKT3 (Brigham and Women’s Hospital)

Also known as oral anti-CD3, is already an FDA-approved drug for the treatment of patients undergoing solid organ transplantation but it has shown up to significantly toxic when administered intravenously. It is also known as Muronomab-CD3, which was the first monoclonal antibody-based therapy for clinical application [191]. In an animal model, OKT3 suppresses autoimmunity. The oral form of OKT3 is being studied in a Phase II clinical trial in combination with omeprazole for treatment of ulcerative colitis but in the animal model, it has been shown to suppress autoimmunity (NCT01287195) [192]. It is also known as Muronomab-CD3 where in the past Muromonab has been already tried as the first monoclonal antibody-based therapy for clinical applications [193].

### 6.20. Foralumab (Tiziana Life Sciences Plc.)

Foralumab is an oral formulation of anti-CD-3 antibody developed for the treatment of autoimmune and inflammatory diseases such as nonalcoholic steatohepatitis (NASH) and nonalcoholic fatty liver disease (NAFLD). A Phase II clinical trial (NCT03291249) conducted by Novimmune was withdrawn at the request of the Ministry of Health [194]. Further clinical trials are planned by the manufacturer [195].

## 7. Conclusions

The use of TPPs as drug candidates has been facilitated by the discovery of recombinant DNA technology and solid-phase protein synthesis, which allowed a large scale of synthesis of TPPs. A variety of peptides and proteins are currently in use for treating different diseases but mostly through the parenteral route, yet the oral route is the most preferred route for administration due to patient compliance and ease of administration. The therapeutic potential of orally delivered TPPs is somewhat hampered by systemic instability in the GI tract and poor absorption through intestinal epithelia. To exert their activity, TPPs must remain stable and active before reaching the systemic circulation. In the last few years, significant efforts were made to enhance the bioavailability of orally administered TPPs. Absorption enhancers, enzyme inhibitors, structural modifications, and carrier systems were developed to tackle systemic instability and increase the bioavailability of TPPs. These strategies have met with initial success but very few of them have yet reached the clinics. An ideal oral formulation must meet all the demands of patients, healthcare providers, and pharmaceutical manufacturers. Furthermore, a thorough understanding of the factors that affect stability in the GI tract and permeation across the intestinal epithelia of TPPs will be essential in developing the ideal TPP formulations for oral delivery, particularly those of children.

## Figures and Tables

**Table 1:** Comparison of pharmacokinetic parameters observed in adults and pediatrics.

Pharmacokinetic Parameter	Adults	Pediatrics
Absorption	Gastric pH: Acidic 2–3.5; Gastric emptying rate: faster than in newborns and neonates.	Gastric pH: alkaline (6–8) in neonates; gastric emptying rate: reduced in infants, takes 6–8 months to reach adult phase [47].
Distribution	The volume of distribution of hydrophilic drugs is high in adults due to high body water content; high plasma protein binding; low permeability of the blood-brain barrier	Low volume of distribution due to low body water content; low plasma protein binding leads to an increase in the unbound fraction of drugs; increased permeability of blood-brain barrier [48].
Metabolism	Hepatic enzymes in the CYP (Phase I) and N-acetyltransferase NAT (Phase II) families contribute significantly to drug metabolism	Low hepatic metabolizing enzyme activity; CYP-450 enzymes are immature in neonates and children [49].
Excretion	Renal clearance is one of the most important paths of drug elimination; kidney function matures with age.	Glomerular filtration rate, renal blood flow, the renal tubular function is much lower (30–40%) in newborns, due to the smaller, underdeveloped kidney in newborns [50].

**Table 2:** Commonly used permeation enhancers for oral delivery of therapeutic peptides and proteins thrombotic thrombocytopenic purpura (TPPs).

Class	Examples	Mechanism of Action
Chelating agents	* 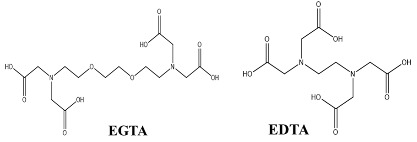 *	They modulate and perturb structural integrity of tight junctions
Surfactants	* 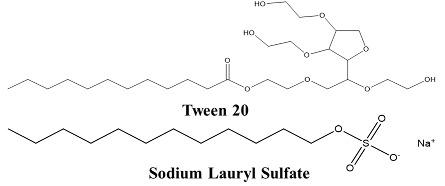 *	Destabilizes both proteins and lipid present at membrane and increase intercellular space for transportation of molecules
Bile salts	* 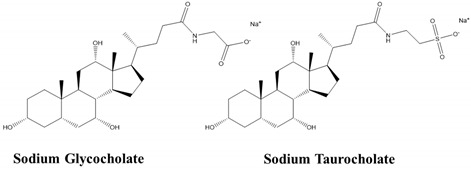 *	They are endogenous surfactant and act above CMC to eliminate membrane lipid components
Fatty acids and their salts	* 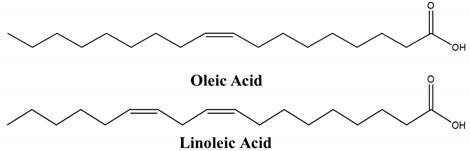 *	They disintegrate the tight junctions and solubilize phospholipid bilayers
